# HPV and HCMV in Cervical Cancer: A Review of Their Co-Occurrence in Premalignant and Malignant Lesions

**DOI:** 10.3390/v16111699

**Published:** 2024-10-30

**Authors:** Rancés Blanco, Juan P. Muñoz

**Affiliations:** 1 Independent Researcher, Av. Vicuña Mackenna Poniente 6315, La Florida 8240000, Chile; 2 Laboratorio de Bioquímica, Departamento de Química, Facultad de Ciencias, Universidad de Tarapacá, Arica 1000007, Chile

**Keywords:** human papillomavirus, human cytomegalovirus, co-infection, oncoproteins, cervical cancer

## Abstract

Cervical cancer remains a significant global health concern, particularly in low- and middle-income countries. While persistent infection with high-risk human papillomavirus (HR-HPV) is essential for cervical cancer development, it is not sufficient on its own, suggesting the involvement of additional cofactors. The human cytomegalovirus (HCMV) is a widespread β-herpesvirus known for its ability to establish lifelong latency and reactivate under certain conditions, often contributing to chronic inflammation and immune modulation. Emerging evidence suggests that HCMV may play a role in various cancers, including cervical cancer, through its potential to influence oncogenic pathways and disrupt host immune responses. This review explores clinical evidence regarding the co-presence of HR-HPV and HCMV in premalignant lesions and cervical cancer. The literature reviewed indicates that HCMV is frequently detected in cervical lesions, particularly in those co-infected with HPV, suggesting a potential synergistic interaction that could enhance HPV’s oncogenic effects, thereby facilitating the progression from low-grade squamous intraepithelial lesions (LSIL) to high-grade squamous intraepithelial lesions (HSIL) and invasive cancer. Although the precise molecular mechanisms were not thoroughly investigated in this review, the clinical evidence suggests the importance of considering HCMV alongside HPV in the management of cervical lesions. A better understanding of the interaction between HR-HPV and HCMV may lead to improved diagnostic, therapeutic, and preventive strategies for cervical cancer.

## 1. Introduction

Cervical cancer is the fourth most frequently diagnosed malignant tumor and also the fourth biggest cause of cancer-related death in women worldwide [1]. In 2020, it was estimated that about 604,127 new cases of cervical cancer and 341,831 deaths were attributable to this malignancy. Cervical cancer is also the leading cause of cancer-related death in 36 countries, mainly in low- and middle-income economies [1]. Despite progress in both screening programs and treatment strategies, cervical cancer still represents an important health problem worldwide. The majority of cervical tumors (≥95%) arise from epithelial cells. Of them, 80–90% are squamous cell carcinomas (SCCs), and 9–14% are adenocarcinomas (AC), while other histological types such as adenosquamous and small cell neuroendocrine carcinomas are minorly represented (<3%) [2,3].

Many risk factors have been associated with cervical cancer development. These risk factors include, but are not limited to, young age at first sexual intercourse, multiple sexual partners, high parity, long-term use of oral contraceptives, a family history of cervical cancer, a diet low in fruits and vegetables, smoking, and socio-economic level, as well as other sexually transmitted infections (e.g., HIV) [4]. However, the most important risk factor for developing cervical SCCs by far is the persistent infection with human papillomavirus (HPV). In fact, HPV infection is detected in 99.7% of cervical SCCs [5]. Despite the introduction of immunization against HPV, about 15.1–45.1% of women still tested positive for HPV DNA [6,7,8]. Moreover, in vaccinated women, an important number of cervical precancerous lesions associated with non-preventable high-risk HPV (HR-HPV) genotypes are diagnosed [9,10].

HPV-induced cervical carcinogenesis is characterized by a stepwise progression from the low-grade squamous intraepithelial lesion (LSIL) to the high-grade squamous intraepithelial lesion (HSIL) and cervical SCC [11]. However, it was reported that 60.0–79.4% of LSILs associated with HPV infection regress to normal cytology, while just 3.6–17.0% of these premalignant lesions progress to HSIL [12,13,14] and potentially cause cancer. Accordingly, it is well accepted that HPV infection is a prerequisite, but it is insufficient for cervical cancer development. In this regard, other host and/or environmental co-factors potentially involved in the development of cervical cancer have been proposed. Among them, the infection with a second virus (co-infection), such as Epstein–Barr virus (EBV) [15], Merkel cell polyomavirus (MCPyV) [16], herpes simplex virus type 2 (HSV2) [17], and human cytomegalovirus (HCMV) [18], is under investigation.

Historically, the role of HCMV in the transformation of cervical epithelial cells and its potential involvement in the development of cervical cancer has been a subject of considerable debate and skepticism among researchers [19,20]. However, emerging evidence suggests that specific HCMV strains, often referred to as high-risk HCMV strains, possess the ability to induce oncogenic phenotypes in epithelial cells, thereby contributing to cancer progression [21,22,23,24]. This review aims to provide an in-depth analysis of the current literature concerning the presence and potential impact of HCMV in squamous intraepithelial lesions (SILs) and cervical cancer, shedding light on the controversial yet evolving understanding of the oncogenic potential of HCMV in cervical carcinogenesis.

## 2. Role of HPV Infection in Cervical Carcinogenesis

**Figure 1:**
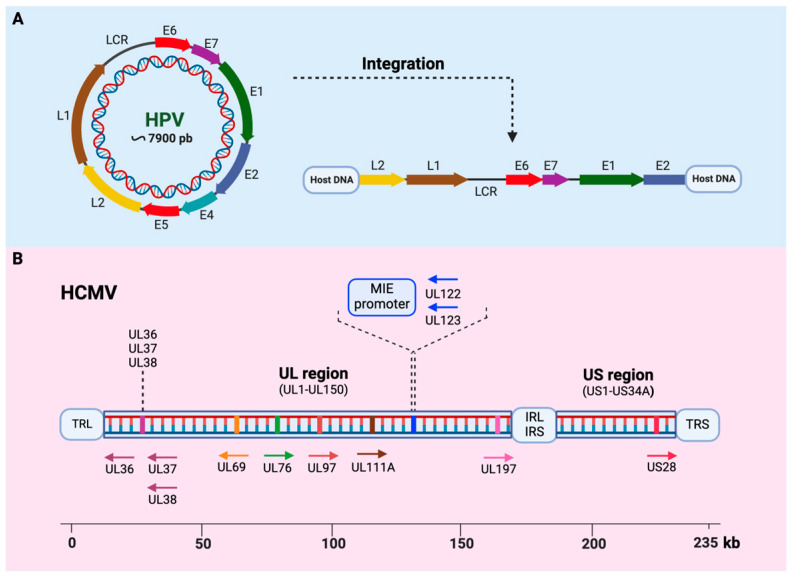
Schematic representation of human papillomavirus (HPV) and human cytomegalovirus (HCMV) genome organization. (**A**) The HPV contains a circular double-stranded DNA genome organized into three regions: the early (E) region, the late (L) region, and the long control region (LCR). The integration of HPV into the host genome induces a disruption or loss of the E2 suppressor gene sequences, which leads to the constitutive expression of E6 and E7 oncogenic proteins. (**B**) The HCMV contains a linear double-stranded DNA genome divided into the unique long (UL) and the unique short (US) regions. These regions are flanked by inverted terminals and internal open reading frame repeats. The relative position and orientation of some key HCMV gene products related to the oncogenic potential of this virus are also shown.

HPV is a non-enveloped virus that belongs to the *Papillomaviridae* family [25]. The viral genome consists of a double-stranded circular DNA with approximately 8 kb in length (7900 bp) surrounded by an icosahedral capsid composed of 72 capsomeres [26]. The HPV genome is divided into three regions: an early region (E), a late region (L), and a long control region (LCR). The early region encodes six non-structural proteins (E1, E2, E4, E5, E6, and E7), while the late region encodes two structural proteins (L1, the major capsid protein, and L2, the minor capsid protein) [27] (Figure 1). The LCR is a non-coding regulatory region involved in controlling early gene expression [28] and is about 1000 base pairs long, located between the L1 and E6 genes [29].

HPV genotyping, based on the L1 DNA sequence, has identified approximately 210 genotypes, categorized by their oncogenic potential. High-risk HPV (HR-HPV) types include HPV16, 18, 31, 33, 35, 39, 45, 51, 52, 56, 58, 59, 66, 68, and 70, while low-risk (LR) types, such as HPV6, 11, 42, 43, and 44, are less associated with cancer development [30,31].

The early proteins E1 and E2 are primarily involved in viral DNA replication and transcriptional regulation [32,33]. E4 contributes to various aspects of the viral life cycle, including release and transmission [34]. The key oncogenic proteins E5, E6, and E7 are responsible for driving HPV-induced carcinogenesis by targeting multiple cellular pathways, leading to unchecked cell cycle progression, apoptosis evasion, angiogenesis, oxidative stress, DNA damage, and immune evasion [35,36].

### 2.1. HPV Lifecycle

HPV invades the basal layer of the cervical stratified epithelium through micro-abrasions that occur during intimate sexual contact [37]. To infect the basal cells, HPV binds to the host extracellular matrix (ECM) mainly through interaction with heparan sulfate proteoglycans (HSPG) [38]. For HPV uptake into the cells, a variety of surface receptors and accessory proteins, including but not limited to tetraspanins, laminin-binding integrins, and growth factor receptors (GFRs), are also required [39,40]. Subsequently, HPV is internalized via a macropinocytosis-like pathway [41], uncoated, and transported into the nucleus [40,42]. In the non-productive phase of the HPV lifecycle, low copies of the viral genome are maintained in episomal status in basal cells [43].

Distinctively, the productive phase of the HPV lifecycle occurs in the suprabasal layers of stratified epithelium, in which differentiated cells have suppressed the DNA replication activity [44]. At this point, HPV E6 and E7 induce the degradation of p53 and retinoblastoma (Rb) tumor suppressor proteins, respectively. This enables the differentiated epithelial cells to maintain their DNA replication machinery [45]. In the spinous layer of stratified epithelium, the HPV genome is amplified to thousands of copies per cell. Afterward, the synthesis of L1 and L2 capsid proteins is triggered, viral genomic DNA is encapsulated, and newly synthesized virions are released from the terminally differentiated keratinocytes [44].

### 2.2. HR-HPV Integration and Oncogenesis

HR-HPV integration into the host genome is a critical step in cervical cancer development, facilitating the persistent expression of viral oncogenes E5, E6, and E7 [46,47,48]. Upon full integration, the virus loses its ability to produce encapsulated circular genomes, halting the production of progeny virions [49]. During this process, the viral genes E1 and E2 are often disrupted, which deregulates viral gene expression [50]. The loss of E2, a repressor of E6 and E7 expression, leads to the continuous production of these oncogenic proteins [33,51]. Once expressed, HPV E6 binds to the E3 ubiquitin-ligase E6-associated protein (E6AP), promoting the proteasomal degradation of the tumor suppressor p53 [52,53]. This degradation impairs the DNA damage response (DDR) mechanisms, allowing cells with damaged DNA to avoid p53-mediated apoptosis [54]. Simultaneously, E7 targets the Rb protein for degradation via the ubiquitin–proteasome pathway, liberating the E2F-responsive genes essential for DNA synthesis [55,56]. E5 also contributes by activating the epidermal growth factor receptor (EGFR) and inhibiting receptor degradation, which promotes cell proliferation [57,58].

HR-HPV E6 also induces chronic oxidative stress, which leads to DNA damage. The spliced isoform E6* contributes to the production of reactive oxygen species (ROS) by downregulating antioxidant enzymes, such as superoxide dismutase 2 (SOD2) and glutathione peroxidase (GPx) [59]. To evade apoptosis induced by this genetic instability, HPV employs a range of anti-apoptotic strategies [52,53,60]. Additionally, E7 oncoprotein targets the DNA repair protein ring finger protein 168 (RNF168), disrupting the host cell’s ability to repair double-strand DNA breaks and increasing chromosomal instability [61]. This further enhances the likelihood of HPV integration into host chromatin [62,63].

The viral integration is also associated with other chromosomal abnormalities, such as polysomies, aneusomies, and an increased number of centrosomes [48,64]. Although HPV integration occurs randomly in the cervical cell genome, frequent integration sites have been identified in HSIL and cervical cancer [65,66]. Moreover, it was identified that HPV integration sites into the human telomerase reverse transcriptase (hTERT) gene lead to the gain of telomeric activity, resulting in cell immortalization [67]. HR-HPV E6 protein also mediates the activation of the catalytic subunit of hTERT, contributing to HPV-infected cell immortalization [68,69].

In summary, HPV plays a crucial role in cervical carcinogenesis by intricately interacting with host cellular mechanisms. The viral oncoproteins E5, E6, and E7 are key drivers of this process, as they disrupt essential cellular pathways, leading to uncontrolled cell proliferation, evasion of apoptosis, and increased genomic instability. These alterations create a permissive environment for the integration of high-risk HPV into the host genome, a pivotal step in the progression toward cervical cancer. This integration, along with the sustained expression of viral oncogenes, underpins the malignant transformation of cervical cells.

## 3. Other Factors Contributing to Cervical Cancer Development

While HPV infection remains the primary cause of cervical cancer, additional factors can influence the progression of HPV-related lesions into malignancy. Several co-infections with sexually transmitted pathogens have been linked to an increased risk of persistent HPV infection and cervical cancer development. These pathogens may affect the natural course of HPV infection by exacerbating inflammation, promoting genomic instability, or modulating immune responses. This section explores how these co-infections act as cofactors in cervical cancer pathogenesis, underscoring their contribution to the complex interplay between HPV and host cells.

### 3.1. Chlamydia Trachomatis (CT)

CT is a common sexually transmitted intracellular pathogen associated with increased susceptibility to HPV infection and persistence [70]. Meta-analyses have demonstrated that CT-positive patients have a significantly higher risk of HPV infection and a prolonged duration of HPV persistence. For instance, a meta-analysis by Liang et al. found that CT-positive individuals had a 3.16-fold higher risk of HPV infection compared to CT-negative individuals [71], while Vielot et al. reported a 3.4-fold increased duration of HR-HPV infection in CT co-infected women [72]. Furthermore, the presence of CT was associated with a higher risk of cervical intraepithelial lesions and progression to cancer, as observed in a study where CT co-infection increased the odds of cervical lesions (OR = 7.3) [73].

Mechanistically, CT infection induces reactive ROS production and DNA damage, which can contribute to oncogenic transformation. In HPV16 E6/E7-immortalized cells, CT infection upregulates the expression of ERK and cyclin E, promoting cell proliferation despite ongoing DNA damage [74]. Moreover, the plasmid-encoded protein Pgp3 in CT inhibits apoptosis via PI3K/AKT signaling, further contributing to cellular survival and proliferation under stress conditions [75]. These findings suggest that CT facilitates both HPV persistence and cervical lesion progression, although further research is needed to confirm this hypothesis.

### 3.2. Genital Warts (GWs) and LR-HPV Strains

GWs, caused primarily by LR-HPV strains such as HPV6 and HPV11, are generally considered non-oncogenic [76,77]. However, studies have indicated that women with GWs are at a higher risk of developing in situ cervical carcinoma compared to the general population [78]. Although LR-HPV infections alone are rarely linked to cervical cancer progression, approximately 30% of GWs present co-infections with HR-HPV strains [79]. This co-infection significantly increases the risk of cervical cancer development, suggesting that HR-HPV co-infections may play a role in exacerbating the risk associated with LR-HPV infections.

### 3.3. Herpes Simplex Virus Type II (HSV-2)

HSV-2 is another sexually transmitted pathogen implicated as a potential cofactor in cervical cancer [80]. Co-infection with HSV-2 and HPV has been shown to increase the risk of cervical cancer. In a study by Bahena-Roman et al., HSV-2-seropositive patients had a 1.7-fold higher risk for HR-HPV infection than HSV-2-negative individuals [81]. Furthermore, women co-infected with HPV and HSV-2 exhibited a significantly higher risk of developing cervical cancer (RR = 3.44) compared to those infected with either virus alone [82].

At the molecular level, HSV-2 has been shown to increase the expression of key HPV oncogenes (E1, E2, and E6), promote the overexpression of survivin (an inhibitor of apoptosis), and enhance the integration of HPV18 DNA into the host genome [83,84]. These processes can drive oncogenesis by disrupting cellular homeostasis, evading apoptosis, and promoting unchecked cellular proliferation. While the exact role of HSV-2 in cervical lesion progression is debated, its potential to initiate or exacerbate early HPV-driven transformation suggests it may contribute to cervical cancer development.

### 3.4. Epstein–Barr Virus (EBV)

Recent studies suggest a potential role for Epstein–Barr virus (EBV) as a cofactor in cervical cancer, particularly in conjunction with HR-HPV co-infection [85,86]. The prevalence of EBV/HPV co-infection is notably higher in cervical squamous carcinomas and HSIL than in low-grade lesions or normal cervical tissues [15,87]. Co-infection with EBV has been associated with a significant increase in the integration of HPV DNA into the host genome, a key event in cervical carcinogenesis [88].

Moreover, in HPV16-positive cervical cancer cells, EBV infection promotes cell proliferation, migration, and epithelial–mesenchymal transition (EMT), contributing to a more aggressive cancer phenotype. The co-expression of EBV LMP1 and HR-HPV E6 proteins has been linked to more advanced disease states, while the EBV BARF1 expression in HeLa cells activates telomerase, a hallmark of cancer cell immortality [86]. Thus, EBV may enhance the oncogenic potential of HPV through multiple molecular mechanisms, although further research is needed to fully elucidate the extent of its contribution to cervical cancer progression.

In summary, while HPV remains the central factor in cervical carcinogenesis, co-infections with sexually transmitted pathogens, such as CT, HSV-2, and EBV, can further exacerbate the progression of HPV-related lesions. These pathogens modulate key cellular and immune processes, promote persistent HPV infection, and contribute to the molecular mechanisms that drive oncogenesis. Building upon this framework, HCMV has emerged as another potential cofactor in cervical cancer. The next section will delve into the role of HCMV and its possible interaction with HPV in promoting malignant transformation.

## 4. HCMV as a Cofactor for Cervical Cancer Development

HCMV (formally called Human Herpervirus-5 or HHV-5) belongs to the Herpesviridae family and the β-herpesvirinae subfamily. HCMV comprises an icosahedral capsid that contains double-stranded genomic DNA of 235 kb in length. It has the largest genome among human herpesviruses. The capsid is surrounded by a tegument with several spike protrusions consisting of viral-encoded glycoproteins. The viral genome is divided into two regions named unique long (UL) and unique short (US). These domains are flanked by a pair of inverted sequences: terminal/internal repeat long (TRL/IRL) and internal/terminal repeat short (IRS/TRS) [89]. The HCMV genome contains an estimated 165–252 open reading frames (ORFs) [90] and encodes 4 long non-coding RNAs [91], about 16 pre-miRNAs, and 26 mature miRNAs [92,93,94].

### 4.1. Prevalence and Impact of HCMV Infection

The prevalence of HCMV infection can reach 90% of the population worldwide [95]. The disease manifestations associated with HCMV infection differ depending on the host immune status. In immunocompetent individuals, HCMV primary infection is usually asymptomatic, although symptoms resembling mononucleosis have been reported [96,97]. On the contrary, in immunocompromised individuals, such as AIDS patients and transplant recipients, HCMV infection causes severe morbidity [98,99,100]. Persistent infection with HCMV induces chronic inflammation, linking virus infection to the development of some cancer types [101].

### 4.2. HCMV Infection Patterns and Viral Lifecycle

HCMV can infect a variety of human cells, such as monocytes, fibroblasts, smooth muscle cells, endothelial cells, and epithelial cells [102]. In epithelial cells, HCMV develops a chronic or “smoldering” program in which infection results in a low level of virus production and can persist for months to years [103]. After cell entry, the viral genome is transported into the nucleus, and the pattern of infection (productive, latent, or chronic) is defined according to some factors, such as cell type and other specificities of virus–host interactions [103].

The productive or lytic phase of HCMV is characterized by a sequential flow of gene expression divided into three groups: immediate early (IE), early (E), and late (L) genes [104,105]. In this phase, the major IE genes (MIE), UL123 (IE1), and UL122 (IE2) play critical roles in subsequent viral gene expression and replication [105] (Figure 1). During latency, HCMV expresses genes including but not limited to UL133-UL13, UL144, the latent unique nuclear antigen (LUNA), the latency-associated viral homolog of IL-10 (LAcmvIL-10), and a small form of the UL123-encoded IE1 protein. Although these genes can be expressed in both latent and lytic infections, in latency, they are detected in the absence of extensive lytic gene expression [103]. The HCMV IE1 and IE2 genes are critical regulators of replication during reactivation from viral latency [106]. However, MIE expression is insufficient to promote complete reactivation and production of progeny viruses [103]. Interestingly, it was reported that cells latently infected with HCMV sporadically express IE genes but fail to produce progeny virus [107]. This fact suggests that HCMV IE proteins could also be involved in other biological functions distinct from those related to viral replication.

### 4.3. Role of HCMV in Cancer Development

The link between HCMV infection and cancer has been the subject of extensive debate [108,109,110]. Studies have shown that the HCMV AD169 strain can induce cervical neoplasia in mice [111]. One hypothesis, known as the “hit and run” mechanism, suggests that HCMV gene expression may initiate oncogenesis, but ongoing viral presence is not required for cancer progression [112]. Recent research has further demonstrated HCMV’s ability to induce oncogenic characteristics in various epithelial cells, including those from the colon [21], breast [22,113], prostate [23], ovary [24], and also in astrocytes [114]. However, the oncogenic potential of HCMV appears to vary among strains. HCMV clinical strains are now thought to be classifiable as either high-risk (HR-HCMV) or low-risk (LR-HCMV), depending on their ability to induce epithelial cell transformation, similar to the classification of HPV types [115,116].

Interestingly, some HCMV clinical strains classified as HR-HCMV, such as HCMV-DB and HCMV-BL, have been shown to upregulate key oncogenic factors, including cyclin D1 and telomerase activity, while also enhancing the expression of Ras and Myc proto-oncogenes. These strains activate critical cancer-related signaling pathways like PI3K/Akt and suppress the tumor suppressor genes p53 and Rb [117,118]. In contrast, low-risk HCMV strains (LR-HCMV), such as HCMV-SD, do not sustain the expression of oncogenic factors like Myc, Akt, or EZH2 in human mammary epithelial cells (HMECs), leading to cell death rather than transformation [117]. These differences among HCMV strains may explain the mixed results seen in studies examining HCMV’s role in cancer development.

HCMV encodes several proteins that interfere with crucial signaling pathways associated with oncogenesis. Key viral proteins include IE1, IE2, pp65, US28, pp71, and others like UL36-38, UL69, UL97, and UL76 [109]. For instance, HCMV IE1 interacts with p107, a Rb-related p107 protein, and alleviates the p107-mediated transcriptional repression of E2F-responsive promoters [119]. Similarly, HCMV IE2 binds to pRb, overcoming Rb-induced cell cycle arrest and driving the cell into replication [120]. In addition, the UL111A protein binds to and disrupts p53-mediated transcription [121], while IE2 enhances cyclin E transcription and blocks p21wafi, which normally inhibits cyclin-Cdk2 activity [122,123]. Another key protein, pp71, targets Rb, p107, and p130 for proteasome degradation, promoting DNA synthesis in otherwise quiescent cells [124,125]. Finally, UL76 induces DNA damage and chromosomal aberrations, further contributing to genomic instability [126].

This complex interplay between the HCMV-encoded proteins and host cellular mechanisms highlights the virus’s potential to promote oncogenesis, particularly through its disruption of tumor suppressor pathways and induction of genomic instability.

### 4.4. Immunomodulatory Role of HCMV

HCMV also plays an important role in modulating the immune system, which has significant implications for the development of cervical cancer. HCMV can evade the host immune responses through the expression of various immunomodulatory proteins, such as UL18 and US28, which mimic host molecules and interfere with immune recognition [127]. These viral proteins can impair the function of natural killer (NK) cells, dendritic cells, and T cells, leading to an immune-tolerant environment that supports viral persistence and chronic inflammation [128]. This immunosuppressive environment may facilitate the survival and proliferation of oncogenic cells, potentially accelerating the progression of cervical cancer. Therefore, the immunomodulatory effects of HCMV may represent another critical factor in its role as a cofactor in cancer development.

Overall, while the role of HCMV as a direct oncogenic virus in cervical cancer remains contentious, accumulating evidence suggests that certain high-risk HCMV strains can act as cofactors in cancer development. These strains not only influence key oncogenic pathways and disrupt tumor suppressor functions but also alter immune surveillance through their immunomodulatory properties, creating an environment conducive to malignant progression. The variability in the oncogenic potential among the HCMV strains highlights the complexity of their involvement in cancer, suggesting that strain-specific differences may account for the inconsistent findings reported in the literature.

## 5. HPV and HCMV Co-Presence in Premalignant Lesions and Cervical Cancer

### 5.1. Prevalence and Risk of HCMV in Cervical Lesions

HCMV infection is frequently detected in cervical samples [129,130,131]. In a systematic review conducted by Marinho-Dias et al., it was shown that HCMV infection in the uterine cervix is more frequent in Africa (61.0%), followed by Asia, Europe, North America, and Oceania, with 26.9%, 16.6%, 12.8%, and 6.29%, respectively [20]. The overall prevalence of HCMV in normal/inflammatory smears from the uterine cervix was 17.4%, while the global rates for this infection were 28.0%, 19.7%, and 44.4% in LSIL, HSIL, and CIS/ICC, respectively [20]. Daxnerova et al. detected HCMV in 2.3% of normal cervical tissues, 6.5% of LSIL samples, and 8.0% of HSIL samples, suggesting an increased risk of lesion progression with HCMV presence. The odds ratio (OR) for the development of LSIL in HCMV-positive samples was 10.62, while for HSIL it was 3.23 [132]. However, no differences in the presence of HCMV infection were obtained between SILs and cervical cancer, suggesting a potential contribution of HCMV to cervical carcinogenesis [133]. (Table 1).

### 5.2. Co-Prescence of HPV and HCMV in Cervical Cancer

Some studies have found an association between HCMV and HPV infections in cervical samples [132,134,135]. For instance, HCMV pp65 protein was evidenced in 23/30 (76.7%) of HPV16-positive cervical carcinomas by immunohistochemistry (IHC), which was significantly increased compared to apparently healthy cervical tissues [136]. It was also reported that women with normal cytology and HPV infections were more likely to be co-infected with HCMV when compared to those without HPV infections (OR = 1.79; 95% CI 0.69–4.63) [137]. Mouglin et al. reported the presence of HCMV in 30/110 (27.3%) of cervical biopsies using in situ hybridization (ISH). Additionally, HCMV was detected in 16/40 (40.0%) of HPV-positive samples, and an association between these viral infections was established (*p* = 0.023) [134]. Moreover, Szostek et al. found the presence of HCMV DNA in 52% of HPV16-positive cervical lesions using PCR, which was increased in SCC compared with SILs (*p* < 0.006) [18]. Shen et al. found HCMV DNA presence in 40/52 (76.9%) of HPV-positive cervical carcinomas by PCR but not in the uterine leiomyoma tissues used as controls. In consequence, the co-presence of HPV and HCMV was associated with an increased risk of cervical cancer (OR = 178; 95% CI 33.8–940.0; *p* = 0.001), which was higher compared to the group of HPV infection only (OR = 9.1; 95% CI 1.61–59.5; *p* = 0.007) [138]. Similar results were reported by Baldauf et al. for HMCV and HPV16/HPV18 co-infection. Specifically, an increased risk for cervical cancer was evidenced in HPV16/HPV18-positive cases associated with HCMV (OR = 25.38; 95% CI: 1.30–495.27; *p* = 0.024) compared to non-infected control patients [139]. Muhsin and Abbas detected HCMV in 16/28 (56%) of cervical squamous carcinoma by ISH, which was statistically higher compared to adenocarcinoma samples (6/22; 12%). Additionally, the authors found HCMV presence in 10/33 (30.3%) of HPV16-positive cervical carcinomas [140] (Table 1).

The presence of HCMV DNA detected by PCR has also been significantly increased in cervical samples with integrated or mixed forms of HPV16 compared to those with the episomal form alone (*p* < 0.005). In fact, the occurrence of HCMV DNA resulted in a 6-fold (OR 6.069; 95% CI: 1.91–19.22; *p* = 0.002) increase in the probability of the occurrence of the integrated/mixed HPV16 genome, suggesting a potential contribution of HCMV to this critical step in the development of cervical cancer [18]. Additionally, the presence of HCMV DNA by PCR was related to an increased rate of lymph node metastasis in HPV16-positive cervical samples compared to HPV16^+^/HCMV^−^ specimens (17/48, 35.4% vs. 30/152, 19.7%; *p* < 0.05). This fact was evidenced for HPV16, but not when other HPV subtypes were analyzed [141]. All these facts together support the potential synergism between HR-HPV and HCMV for cervical cancer development. However, a lack of relation between HCMV/HPV co-presence and the risk of cervical cancer development was also reported [19,130,131,142,143,144,145,146]. (Table 1)

### 5.3. HCMV in HPV-Negative Cervical Lesions

Interestingly, HCMV has been detected in HPV-negative cervical lesions. Mougin et al. reported that 20% of HPV-negative cervical abnormalities were also positive for HCMV [134], and Broccolo et al. found HCMV in 36.4% to 75.0% of HPV-negative samples, including normal, LSIL, and HSIL cases [130]. Similarly, Grce et al. reported the presence of HCMV DNA in 4/68 (5.9%) of the HPV-negative SILs [131]. HCMV infection was also detected in 21/91 (23.1%) of HPV-negative cervical carcinomas by PCR [141]. In addition, Han et al. reported HCMV infection in 5/9 (55.6%) of HPV-negative SCC using the same method [143].

Although some studies found no increased risk for cervical cancer in HPV-negative lesions with HCMV [129,138], the presence of HCMV in HPV-negative cases indicates its potential involvement in cervical abnormalities, independent of HPV infection. However, the findings related to HCMV infection in HPV-negative SILs and cervical carcinoma should be interpreted cautiously. The limited number of cases where HPV was not detected may be attributed to inadequate sample preservation or challenges in PCR amplification of the HPV L1 gene fragment due to viral integration [5,147]. As a result, the global prevalence of co-infection with HPV and HCMV in cervical cancer might be underestimated.

### 5.4. Proposed Model of HR-HPV and HCMV Co-Infection in Cervical Cancer

**Figure 2:**
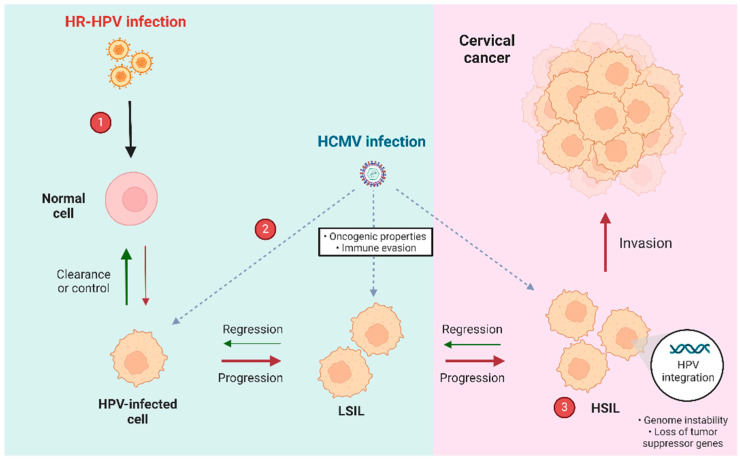
Hypothetical model of uterine cervix carcinogenesis induced by HR-HPV and HCMV co-infection. 1: HR-HPV infects cervical epithelial cells. 2: HR-HPV-infected cells become susceptible to the HCMV second infection, which could contribute to the progression from low-grade intraepithelial lesion (LSIL) to high-grade intraepithelial lesion (HSIL). 3: Genome instability induced by virus oncoproteins in co-infected dysplastic cells could favor HR-HPV integration into the host genome, a crucial step in cervical cancer development.

Based on the available evidence, we propose a model to illustrate the potential impact of co-infection with HR-HPV and HCMV in the development of cervical cancer. In this model, HR-HPV is hypothesized to initiate the transformation of normal cervical cells, which may either clear the infection or progress into a state of persistent HPV infection. When co-infection with HCMV occurs, the oncogenic processes initiated by HR-HPV are likely exacerbated. HCMV may contribute to these processes by promoting immune evasion, allowing the progression from low-grade LSIL to more severe HSIL. (Figure 2).

The combined effects of HR-HPV and HCMV co-infection could amplify key cancer-promoting mechanisms, including increased genomic instability, the inactivation of tumor suppressor functions, and the enhanced invasive potential of the infected cells. This synergistic interaction between the two viruses may accelerate cervical cancer progression. As a result, this dual infection model highlights the potential clinical significance of HCMV as a cofactor in HPV-mediated carcinogenesis and underscores the need to consider both infections when developing management and prevention strategies for cervical cancer.

**Table 1:** HPV and HCMV co-presence in epithelial cells from uterine cervix.

Ref.	Sample	HPV		HCMV		HCMV in HPV-Positive Samples
		Methods	Results	Methods	Results	
[132]	Cytology	PCR	No SIL = 138/266 (51.9%) CL ^a^ = 196/316 (62.0%) LSIL = 109/154 (70.8%) HSIL = 193/250 (77.2%)	PCR	No SIL = 6/266 (2.3%) CL ^a^ = 50/316 (15.8%) LSIL = 10/154 (6.5%) HSIL = 20/250 (8.0%)	No SIL = 5/138 (3.6%) CL ^a^ = 27/196 (13.8%) LSIL = 7/109 (6.4%) HSIL = 11/193 (5.7%)
[133]	FFPE	PCR	Cervicitis = 15/38 (39.5%) CIN I–II = 7/11 (63.6%) CIN III = 5/14 (35.7%) CC ^b^ = 23/35 (65.7%)	PCR	Cervicitis = 2/38 (5.3%) CIN I–II = 4/11 (36.4%) CIN III = 5/14 (35.7%) CC ^b^ = 12/35 (34.3%)	Cervicitis = 2/15 (13.3%) CIN I–II = 3/7 (42.9%) CIN III = 2/5 (40.0%) CC ^b^ = 8/23 (34.8%)
[136]	FFPE	IHC for HPV16	- ^c^	IHC for pp65	Normal = 1/10 (10.0%) CC = 23/30 (76.7%)	CC = 23/30 (76.7%)
[134]	FFPE	ISH	CL ^a^ = 56/131 (42.7%)	ISH	CL ^a^ = 30/110 (27.3%)	CL ^a^ = 16/56 (28.6%)
[18]	Cytology	PCR for HPV16	- ^c^	PCR	LSIL = 10/27 (37.0%) HSIL = 3/10 (30.0%) SCC = 18/23 (78.3%)	LSIL = 10/27 (37.0%) HSIL = 3/10 (30.0%) SCC = 18/23 (78.3%)
[138]	Frozen tissues	PCR	Leiomyoma ^d^ = 3/55 (5.5%) SCC = 52/78 (66.7%)	PCR	Leiomyoma ^d^ = 36/55 (65.5%) SCC = 59/78 (75.7%)	Leiomyoma ^d^ = 0/3 SCC = 40/52 (76.9%)
[139]	FFPE	PCR for HPV16/18/6/11	Normal = 7/33 (21.2%) HSIL = 18/21 (85.7) SCC = 15/20 (75.0%)	PCR	Normal = 7/33 (21.2%) HSIL = 5/21 (23.8%) SCC = 5/20 (25.0%)	Normal = 1/7 (14.3%) HSIL/SCC = 7/33 (21.2%)
[140]	FFPE	ISH for HPV16	ADC = 11/22 (50.0%) SCC = 22/28 (78.6%)	ISH	ADC = 6/22 (27.3%) SCC = 16/28 (57.1%)	ADC/SCC = 10/33 (30.3%)
[141]	Frozen tissues	PCR	SCC = 342/433 (79.0%)	PCR	SCC = 113/433 (26.1%)	SCC = 92/342 (26.9%)
[146]	Frozen	PCR for HPV16, 18, 31, 33, 52, and 58	CC ^d^ = 89/103 (86.4%)	PCR	CC ^d^ = 4/103 (3.9%)	CC ^d^ = 3/89 (3.4%)
[130]	Cytology	PCR for HR-HPVs	Normal = 6/66 (9.1%) ASCUS = 17/39 (43.6%) LSIL = 30/46 (65.2%) HSIL = 53/57 (93.0%)	PCR	Normal = 48/66 (72.7%) ASCUS = 12/39 (30.8%) LSIL = 30/46 (65.2%) HSIL = 47/57 (82.5%)	Normal = 4/6 (66.7%) ASCUS = 4/17 (23.5%) LSIL = 21/30 (70.0%) HSIL = 44/53 (83.0%)
[19]	Cytology	PCR	Normal/ASCUS = 11/52 (21.2%) LSIL = 2/4 (50.0%) HSIL = 4/4	PCR	Normal/ASCUS = 20/52 (38.5%) LSIL = 1/4 (25.0%) HSIL = 2/4 (50.0%)	Normal/ASCUS = 5/11 (45.5%) LSIL = 0/2 HSIL = 2/4 (50.0%)
[131]	Cytology	PCR	LSIL = 24/37 (64.9%) HSIL = 35/128 (27.3%)	PCR	LSIL = 3/37 (8.1%) HSIL = 6/128 (4.7%)	SILs = 5/59 (8.5%)
[143]	FFPE	PCR	SCC = 32/42 (76.2%)	PCR	SCC = 27/42 (64.3%)	SCC = 23/32 (71.9%)
[144]	Cytology	PCR	Normal/Inflamed = 44/201 (21.9%) LSIL = 37/56 (62.5%) HSIL = 70/107 (65.4%) SCC = 21/24 (87.5%)	PCR	Normal/Inflamed = 17/201 (8.5%) LSIL = 9/56 (16.1%) HSIL = 11/107 (10.3%) SCC = 0/24	Normal/Inflamed = 8/44 (18.2%) LSIL = 6/37 (16.2%) HSIL = 8/70 (11.4%) SCC = 0/21
[148]	FFPE	PCR for HPV16	LSIL = 9/22 (40.39%) HSIL = 37/55 (67.3%) SCC = 19/25 (76.0%)	PCR	LSIL = 1/22 (4.5%) HSIL = 4/55 (7.3%) SCC = 2/25 (8.0%)	LSIL = 0/9 HSIL = 2/37 (5.4%) SCC = 2/19 (10.5%)

^a^ Cervical lesions (CL), HPV-induced cytopathic changes; ^b^ Cervical carcinoma (CC), including squamous cell carcinoma (SCC) and other types of cervical tumors, such as adenocarcinoma (ADC) and adenosquamous carcinoma (ADS); ^c^ study performed only in HPV16-positive cases; ^d^ Uterine leiomyoma used as control group; CIN, Cervical intraepithelial neoplasia; SIL, Squamous intraepithelial lesion; LSIL, Low-grade squamous intraepithelial lesion; HSIL, High-grade squamous intraepithelial lesion; ASCUS, Atypical squamous cells of undetermined significance; FFPE, Formalin-fixed and paraffin-embedded tissues; ISH, in situ hybridization; IHC, Immunohistochemistry.

## 6. Conclusions

The available evidence suggests that HCMV may play a significant cofactor role in HPV-driven cervical carcinogenesis. The frequent detection of HCMV in cervical lesions, particularly in those co-infected with HPV, points to a potential synergistic interaction that could enhance the oncogenic processes, including immune evasion and genomic instability, contributing to the progression from LSIL to HSIL and ultimately cervical cancer. Although the exact mechanisms of this interaction require further research, the findings underscore the need to consider HCMV alongside HPV in clinical assessments and preventive strategies. Recognizing the impact of dual HR-HPV/HCMV infection may improve the understanding of cervical cancer pathogenesis and lead to more effective diagnostic, therapeutic, and preventive approaches.

## Data Availability

Not applicable.
